# Angular Deformities of the Lower Limb in Children

**DOI:** 10.5812/asjsm.34871

**Published:** 2010-03

**Authors:** Ramin Espandar, Seyed Mohammad-Javad Mortazavi, Taghi Baghdadi

**Affiliations:** 1 Department of Orthopedic Surgery, Tehran University of Medical Sciences, Tehran, IR, Iran; 2 Sports Medicine Research Center, Tehran University of Medical Sciences, Tehran, IR, Iran

**Keywords:** Genu varum, Genu valgum, Angular deformity of the limbs, Children, Sport

## Abstract

Angular deformities of the lower limbs are common during childhood. In most cases this represents a variation in the normal growth pattern and is an entirely benign condition. Presence of symmetrical deformities and absence of symptoms, joint stiffness, systemic disorders or syndromes indicates a benign condition with excellent long-term outcome. In contrast, deformities which are asymmetrical and associated with pain, joint stiffness, systemic disorders or syndromes may indicate a serious underlying cause and require treatment.

Little is known about the relationship between sport participation and body adaptations during growth. Intense soccer participation increases the degree of genu varum in males from the age of 16. Since, according to some investigations, genu varum predisposes individuals to more injuries, efforts to reduce the development of genu varum in soccer players are warranted. In this article major topics of angular deformities of the knees in pediatric population are practically reviewed.

## INTRODUCTION

A lthough from clinical experiences an association between intense sports activities like soccer players and genu varum seems evident, today no scienti?c data regarding this association are available. Chantraine ^[1]^ hypothesized that the high amount of stress and strain imposed on a joint during growth and adolescence through intensive practice of a sport may contribute to a growth deformity. This possibility is pertinent, but not yet investigated for the development of genu varum and playing soccer. However, several studies showed that the presence of genu varum predisposes an individual to various injuries. For example, genu varum has been associated with the deterioration of the articular cartilage in the knee's medial tibiofemoral compartment both to experimentally induced osteoarthritis and as a risk factor for osteoarthritis in a patient cohort ^[2–4]^.The presence of genu varum alters the forces at the knee so that the line of force shifts farther medially from the knee joint center intensifying the medial compartment load and creating a medial joint reaction force that is nearly three and a half times that of the lateral compartment ^[5]^. In addition to the development of tibio femoral osteoarthritis, the presence of genu varum seems also to predispose subjects to the occurrence of injuries at the patellofemoral joint. Several studies have identi?ed the presence of genu varum as a risk factor for the development of the patellofemoral pain syndrome in athletes ^[6–10]^.

Since the knee malalignment is important in athletes, the development of normal tibiofemoral angle and common angular deformities in children and adolescents are explained in this article.

## NATURAL HISTORY

Genu varum (bow legs) and medial tibial torsion are normal in newborn and infants and maximal varus is present at 6 to 12 months of age. With normal growth, the lower limbs gradually straighten with a zero tibiofemoral angle by 18 to 24 months of age (when the infant begins to stand and walk). With further normal development, knees gradually drift into valgus (knock-knee). This valgus deformity is maximal at around age 3–4 years with an average lateral tibiofemoral angle of 12 degrees ^[11]^. Finally the genu valgum spontaneously correct by the age of 7 years to that of the adult alignment of the lower limbs of 8 degrees of valgus in the female and 7 degrees in the male. The greater degree of valgus in females may be due to their wider pelvis.

Extrinsic and intrinsic factors may interfere with this normal angular alignment of the lower limbs.

## PERSISTENT GENU VARUM IN THE OLDER CHILD

Bowlegs after 2 years of age are considered abnormal. It may be due to persistence of severe physiologic bowlegs (the most common etiology), a pathologic condition, or a growth disorder.

### Assessment

***History:*** Family history of bowlegs or other limb deformities and the presence of short stature may indicate the possibility of bone dysplasia or a generalized growth disorder. Physiologic genu varum improves with growth, whereas pathologic bowing of the legs increases with skeletal growth; therefore it seems important to ask the parents about:

1. When they first noticed the deformity in the child.
2. Were the legs bowed at birth and in infancy, or did the bowlegs develop later on when the child started walking?
3. Is the deformity improving, staying the same, or increasing in severity?
4. When did the child begin to stand and walk? Children with tibia vara (Blount's disease) are early walkers. Inquire as to the previous treatment and response to it.

***Etiologic factors*:** The physician should be aware of the dietary and vitamin intake of the patient ^[12]^ and also should consider any allergy to milk, history of trauma or infections and inquire the possibility of exogenous metal intoxication, specifically lead and fluoride ^[13]^.

***Examination*:** Short stature suggests the possibility of vitamin D refractory (hypophosphatemic) rickets ^[14]^ or bone dysplasia, such as achondroplasia or metaphyseal dysplasia ^[15]^.

In stance and supine the physician should measure the distance between the femoral condyles at the joint level with the ankles just touching each other. Ruling out the deformity of the feet especially pes or metatarsus varus or valgus which may represent torsional deformity of the limb is mandatory ^[16]^.

The site of varus angulation should be determined. In physiologic genu varum there is a gentle curve involving both the thigh and the leg with more pronounced bowing in the lower third of the femur and at the juncture of the middle and upper thirds of the tibia ^[17]^; whereas in ligamentus hyperlaxity it is at the knee joint. In Blount's disease it is commonly at the proximal tibial metaphysis with an acute medial angulation immediately below the knee and in the congenital familial form of tibia vara it is at the lower tibia at the junction of the middle and the lower thirds ^[18]^. In the very rare distal femoral vara the site of angulation is in the distal femoral metaphysis. When the lower tibiae are the sites of varus angulation, the upper tibial segment is straight and the lower segment angulated.

Next, inspect the gait and determine the foot progression angle; in genu varum the foot progression angle may be medial or normal. When laxity and incompetence of the lateral collateral ligament of the knee are present, the fibular head and upper tibia shift laterally during gait; whereas, in physiologic bowlegs there is no such lateral thrust ^[19]^.

It is important to assess symmetry of involvement. In physiologic genu varum and congenital tibia vara it is usually bilateral and symmetric, whereas in Blount's disease it may be unilateral or bilateral, and when both tibiae are involved, the degree of affection is often asymmetric.

Measure both the actual and apparent limb lengths. In Blount's disease and in congenital longitudinal deficiency of the tibia ^[20]^, the involved or more severely affected limb is shorter than the contralateral one; in physiologic genu varum the lower limb lengths are even.

In the medially bowed leg, determine the level of the proximal fibula in relation to that of the tibia. Normally the upper border of the proximal fibular epiphysis is in line with the upper tibial growth plate – well inferior to the joint horizontal orientation line; whereas Blount's disease, congenital longitudinal deficiency of the tibia, and achondroplasia demonstrate relative overgrowth of the fibula, and the fibular epiphysis is more proximal, near the joint line ^[21]^.

Palpate the epiphysis of the long bones at the ankles, knees, and wrists. In rickets (vitamin D refractory or vitamin deficiency) they are enlarged. Inspect the thoracic cage. Is there a “rachitic rosary” of the ribs, pectus carinatum deformity, or Harrison's groove?

***Imaging*:** Take radiograms when:

1. A child is 3 years and older and the varus deformity is not improving or is getting worse,
2. The medial bowing is unilateral or asymmetric,
3. The site of varus angulation is acute in the proximal tibial metaphysis immediately below the knee,
4. The possibility of a pathologic condition is suggested by other clinical findings. The clinical stigmata suggesting pathologic genu varum are short stature (bone dysplasia), enlarged epiphysis and physis (rickets), history of trauma or infection (meningococcemia) ^[22]^, short tibia and relatively long fibula, and history of possible metal intoxication (lead or fluoride).

Standing long films (AP and lateral) should be made to include the hips, knees, and ankles. Proper positioning is important – knees straight and patella facing forward.

The growth plates of the distal femur and proximal and distal tibia should be considered carefully. In physiologic genu varum they are normal. In rickets the physes are markedly thickened, the physeal borders of the epiphyses are frayed with a brush-like pattern of the bone trabeculae, the epiphyses are enlarged, the bone trabeculae are coarse, and the cortices of the diaphyses of the femurs and tibiae show decreased bone density ^[23]^.

Then, the epiphyses, metaphyses, and diaphyses should be inspected. In physiologic genu varum the bone seems normal without any sign of bone dysplasia. The medial bowing of the lower limb is a gentle curve, taking place at the junction of the middle and the proximal thirds of the tibiae and the distal thirds of the femurs. The horizontal joint lines of both the knee and ankle are tilted medially.

Measure the metaphyseal-diaphyseal angle. In the physiologic genu varum it is less than 11 degrees, whereas in tibia vara it is greater than 11 degrees ^[24]^.List of conditions that cause pathologic tibia vara is given in Table 1.

**Table 1:** Skeletal affections that present as bowlegs

Apparent genu varum Physiologic genu varum Congenital familial tibia vara Tibia vara (Blount's disease) Asymmetric growth arrest of medial part of distal femur and proximal tibia due to infection, fracture, or tumor Rickets – vitamin D deficiency or refractory (hypophosphatemia) Bone dysplasia, such as achondroplasia and metaphyseal dysplasia Fibrocartilagenous dysplasia Congenital longitudinal deficiency of the tibia with relative overgrowth of the fibula Lead or fluoride intoxication

### Treatment

In physiologic genu varum education and assurance of the parents is important and just follow its natural course by reassessing the child in 6 months. Orthopedic shoes are not effective in its prevention or management ^[25]^.

Metabolic deformities such as rickets could simply be corrected with medical treatment, i.e. calcium and vitamin D supplements.

When severe genu varum is associated with severe medial tibial torsion and the metaphyseal-diaphyseal angle is 11 degrees or greater, a Denis Browne splint is prescribed with the feet (shoes) rotated laterally and with an 8 to 10-inch bar between the shoes. This is ordinarily worn only at night for a period not more than 3 to 6 months in order to correct excessive medial tibial torsion ^[26]^.

In the adolescent with severe genu varum with marked malalignment of the mechanical axis of the lower limbs, occasionally osteotomy of the tibia or hemiepiphysiodesis of the distal femur and/or proximal tibial physis is indicated to correct the deformity ^[27]^. It is difficult to calculate the exact age for hemiepiphysiodesis. Stapling is preferred by some authors ^[28]^.

## PERSISTENT EXAGGERATED GENU VALGUM IN THE OLDER CHILD AND ADOLESCENT

Exaggerated genu valgum up to 7 years of age is physiologic and not pathologic ^[29]^. The problem is the adolescent or the child over 8 years of age who present with moderate to severe knock-knees. The patient complains of pain in the thigh and/or calf and easy fatigability, the child walks with his knees rubbing together, feet apart and one leg swinging around the other. Frequently, parents are concerned with this form of gait. Due to malalignment and an increased Q angle of the quadriceps extensor mechanism, the patella subluxates laterally; hence, the patellofemural joint seems to be unstable. The shoes shows medial collapse of the upper parts and it is the result of abnormal weight-bearing forces on the ankle and foot ^[30]^. The parents seek active treatment and commonly believe that the deformity will result degenerative, crippling arthritis of the knee ^[31]^. In order to manage the problem properly, first we should determine the cause of abnormal genu valgum by careful history taking, physical examination, and appropriate imaging studies. The various causes of genu valgum are listed in Table 2.

**Table 2:** Various causes of genu valgum

Developmental – physiologic, no intrinsic bone disease or congenital anomaly Congenital – due to longitudinal deficiency of the fibula Iliotibial band contracture Trauma Malunion of fracture Growth stimulation by greenstick fracture of the proximal tibial metaphysic Asymmetric growth arrest due to fracture-separation involving the lateral segment of the upper tibial physis or distal femoral physis Infection – causing asymmetric growth disturbance Arthritis of knee – rheumatoid, hemophylia Bone dysplasia – Morquio's syndrome, Ellis-Van Creveld syndrome, Ollier's disease(multiple enchondromatosis), multiple hereditary exostosis, metaphyseal dysplasia, multiple epiphyseal dysplasia Osteogenesis imperfecta Metabolic bone disease, particularly

### Assessment

***History:*** The presence of positive family history and short stature in other members of the family will suggest the presence of bone dysplasia, such as multiple epiphyseal dysplasia, multiple metaphyseal dysplasia, multiple enchondromatosis (Ollier's disease), multiple hereditary exostosis, Ellis Van Creveld syndrome ^[32]^, or Morquio's disease ^[33]^. History of swollen and hot knees indicates rheumatoid arthritis ^[34]^. With increased circulation in the knee the tibia overgrows relative to the fibula. In congenital longitudinal deficiency of the fibula, genu valgum is common ^[35]^.

***Examination:*** For assessment of short stature and bony dysplasia the standing and sitting height of the patient should be measured ^[36]^. Inspect the alignment of the lower limb in stance. Measure the degree of genu valgum with a goniometer on the lateral side of the thigh-leg, the distance between the medial maleolli with the knee just touching. Genu requrvatum, if present, causes an apparent increase in the degree of deformity ^[37]^.

Asymmetry or unilateral involvement of the knee is suggestive of pathologic genu valgum. In walking, there is protective toeing-in to shift the foot medially so that the center of gravity falls in the center of the foot ^[38]^.

Ligamentous laxity may be the cause of knock-knees; therefore one should determine the stability of the collateral and cruciate ligaments of the knee.

The site of valgus angulation should be determined. Tibia valga, or greenstick fracture of the medial part of the proximal tibial metaphysic cause genu valgum at the proximal tibia ^[39]^ whereas in ligamentus hyperlaxity, congenital longitudinal deficiency of the fibula, or rheumatoid arthritis of the knee, it is at the knee and in metabolic bone disease and bone dysplasia at the distal femur or in both femur and the tibia. Tibia valga is usually associated with excessive lateral tibiofibular torsion thus the degree of tibial torsion should be assessed ^[40]^.

Iliotibial band contracture may cause tibia valga and its presence should be ruled out by an Ober test ^[41]^. Exostosis and lower limb length inequality should be assessed by careful palpation of the epiphysis and metaphysis and limb length measurements, respectively ^[42]^.

*Imaging findings*: In developmental genu valgum the epiphysis, physis, and metaphysis are normal. The horizontal axis of the knees and ankles is tilted laterally. No intrinsic bone disease is present.

In pathologic genu valgum the radiographic features are usually characteristic, and diagnosis is readily made. When an osseous bridge across the lateral physis of the distal femur and proximal tibia is suspected, MRI should be performed. Bone age should be determined if a hemiepiphysiodesis is being planned ^[43]^.

### Treatment

Special shoes are ineffective in prevention or treatment of genu valgum. If the feet are in valgus and foot strain is a complaint, foot orthotics, such as University of California Biomechanics Laboratory (UCBL) orthotics, are appropriate to support the foot. They do not correct the genu valgum but relieve foot strain, easy fatigability, and foot-calf pain ^[44]^. When the iliotibial band is contracted, passive stretching exercises for its stretching should be done. This may relief valgus deforming force of the knee.

The role of orthotics to control or correct genu valgum has not been proven and is controversial. Some authors do not recommend them. According to the body of literature, the only indication for a Knee Ankle Foot Orthosis (KAFO) is to support the knee ligaments and prevent them from overstretching. It is used in pathologic genu valgum.

In the adolescent with severe genu valgum and marked mechanical axis deviation, surgical correction is indicated. Two methods of surgical management are available:

1. Hemiepiphysiodesis is done by stapling or fusing the medial part of the distal femoral and/or proximal tibial growth plates. Appropriate time is crucial. Despite all precautions, one may end up with overcorrection or undercorrection. Some authors prefer stapling over epiphysiodesis because it allows a certain amount of flexibility of timing ^[45]^.
2. Osteotomy of the distal femur and proximal tibia is performed in the skeletally mature patient. Neurovascular structures, particularly the common peroneal nerve and tibial vessels, are at definite risk for injury. Gradual correction with an external fixator lessens the degree of neurovascular change ^[46–48]^.

## CONCLUSION

Angular deformities of the lower limbs are common during childhood and usually make serious concern for the parents. Most commonly these deformities represent normal variations of the growth and development of the child and needs no treatment except for observation and reassurance of the parents. Despite of the benign nature of physiologic or exaggerated physiologic genu varum and genu valgum, most of the pathologic causes need proper management by an orthopedic surgeon; hence, the importance of careful evaluation of the patients and determination of these pathologic causes is evident.
